# Coding Gaps and Missed Opportunities: How Billing Practices May Improve Gastric Cancer Detection in the United States

**DOI:** 10.14309/crj.0000000000002086

**Published:** 2026-04-10

**Authors:** Sneh Sonaiya, Hareesha Rishabh Bharadwaj, Hema Sameera Pinnam, Vineet Chandak, Harshita Malik, Hassam Ali, Dushyant Singh Dahiya

**Affiliations:** 1 Department of Internal Medicine, University of Nevada, Las Vegas, NV; 2 Department of Internal Medicine, Royal Stoke University Hospital, University Hospitals of North Midlands NHS, Stoke-on-Trent, UK; 3 Department of Internal Medicine, Jagadguru Sri Shivarathreeshwara Medical College, Mysuru, India; 4 Department of Internal Medicine, Seth GS Medical College and KEM Hospital, Mumbai, India; 5 Department of Internal Medicine, Kasturba Medical College, Manipal, India; 6 Department of Gastroenterology, Hepatology and Nutrition, East Carolina University/Brody School of Medicine, Greenville, NC; 7 Division of Gastroenterology, Hepatology and Motility, The University of Kansas School of Medicine, Kansas City, KS

## INTRODUCTION

Gastric cancer (GC) remains a major gastrointestinal malignancy in the United States, with an annual incidence of approximately 7.3 per 100,000 men and women.^1^ Despite its relatively lower incidence compared with other gastrointestinal cancers, GC accounts for nearly 1.7% of all cancer-related deaths in the United States, with an estimated 5-year survival rate of only 37.9%.^1^ Poor 5-year survival outcomes of GC in the United States are driven in part by diagnosis of GC at advanced stages, when the curative options become limited. Furthermore, the total financial burden of GC in the United States was estimated to be ∼$2.31 billion in 2020.^2,3^

Globally, the burden of GC is substantially higher, with nearly 1 million new cases and more than 650,000 deaths annually.^4^ The disease is disproportionately prevalent in East and Central Asia, South America, and Eastern Europe.^5^ *Helicobacter pylori* infection is a well-established risk factor for GC, and primary prevention strategies have largely focused on *H. pylori* detection and eradication. Secondary prevention relies on endoscopic screening and surveillance of premalignant conditions such as gastric intestinal metaplasia, which enables detection of early-stage disease when curative treatment is possible.

## LESSONS FROM HIGH-INCIDENCE COUNTRIES: SOUTH KOREA's NATIONAL CANCER SCREENING PROGRAM

Several countries with a high incidence of GC, including Japan and South Korea, have implemented national screening programs with demonstrable success. In South Korea, the National Cancer Screening Program has provided biennial GC screening for adults aged 40 years and older since 1999. Data from this program show substantial improvements in outcomes, with 5-year relative survival increasing from 43.9% in the mid-1990s to 77.5% between 2015 and 2019.^6,7^ Over the same period, the proportion of early GC diagnoses more than doubled, rising from 28.6% to 63.6%, highlighting the impact of systematic screening and early detection.^6,7^

In contrast, the United States lacks a formal GC screening strategy, despite a growing population at elevated risk. According to the 2020 U.S. Census data, more than 40% of the U.S. population identifies as non-White, with many individuals originating from regions with high GC incidence.^8^ Korean Americans aged 50 years and older have been shown to have a 12- to 14.5-fold higher risk of noncardia GC compared with non-Hispanic White individuals, with similar patterns observed among other immigrant populations from high-incidence regions.^7,9^ Yet, GC screening remains inconsistently implemented, leaving a significant care gap for these high-risk groups, particularly first-generation immigrants.

Recognizing this disparity, the American Gastroenterological Association has issued a conditional recommendation to consider GC screening in individuals aged 45 years and older who are at increased risk, including early-generation immigrants from high-incidence regions, individuals with a first-degree relative with GC, or racial and ethnic groups with established moderate to high incidence of GC.^10^ Despite this guidance, real-world uptake of GC screening in the United States remains limited.

## WHY TIME SPENT DURING ESOPHAGOGASTRODUODENOSCOPY MATTERS

Multiple studies have demonstrated that the quality and thoroughness of esophagogastroduodenoscopy (EGD) directly influence lesion detection. Increased inspection time during EGD has been associated with higher detection rates of premalignant and malignant gastric lesions.^11^ In a study by Teh et al,^12^ endoscopists with mean examination times exceeding 7 minutes identified significantly more high-risk gastric lesions compared with faster endoscopists. Furthermore, studies have shown that adherence to the updated Sydney system biopsy protocol improves the diagnostic yield of gastric preneoplastic conditions such as gastric intestinal metaplasia.^13^ Accordingly, effective GC detection requires systematic mucosal evaluation and adherence to standardized biopsy protocols, such as the updated Sydney system—processes that often necessitate additional inspection time beyond that of routine EGD.

Recently published endoscopy quality indicators emphasized the importance of systematic mucosal inspection and adherence to standardized gastric biopsy protocols in patients at risk for GC.^14^ In particular, systematic gastric biopsy sampling is recommended for patients with gastric premalignant conditions or those at increased risk for GC, including individuals with a family history of GC or immigrants from high-incidence regions.^15^ Furthermore, endoscopic biopsy rate has also been proposed as a quality indicator, and studies have shown that endoscopists with higher gastric biopsy sampling rates have a lower risk of missed GCs than those with lower biopsy sampling rates.^14,16^ These findings further highlight the importance of adequate examination time and appropriate scheduling to ensure high-quality endoscopic evaluation in busy clinical practice.

## HOW BILLING AND REIMBURSEMENT AFFECT GC SCREENING

The financial implications of GC diagnosis further highlight the potential value of early detection strategies. The mean total GC-related cost per patient during the postindex follow-up period has been estimated at ∼$70,808 ± $56,620, reflecting the substantial economic burden associated with GC treatment.^17^ In contrast, the national average Medicare reimbursement for a diagnostic EGD is ∼$1,036, suggesting that screening endoscopy represents a relatively low-cost intervention.^18^ Importantly, U.S.-based cost-effectiveness studies have demonstrated that risk-based GC screening with EGD may be economically justified in several high-risk populations—including Asian, Black, and Hispanic individuals, immigrants from high-incidence regions, and those with a family history of GC—with incremental cost-effectiveness ratios ranging from $76,200 to $99,500 per quality-adjusted life-year gained.^19^ Modeling studies further suggest that EGD screening at age 55 with conditional 5-year surveillance could prevent 1 GC case for every 343 EGDs performed and 1 GC-related death for every 431 EGDs performed, highlighting the potential population-level impact of targeted screening strategies.^19^

Additional studies have suggested that combining EGD with screening colonoscopy may represent an efficient screening strategy. A U.S. modeling study demonstrated that bundling EGD with screening colonoscopy at age 50 may be cost-effective in high-risk racial and ethnic populations.^20^ Similarly, a European cost-effectiveness analysis conducted from the perspectives of Italy and Portugal suggested that performing screening EGD concurrently with screening colonoscopy once per decade may also be a cost-effective strategy for GC detection compared with no screening.^15^

Notably, reimbursement trends for upper endoscopy have moved in the opposite direction. A recent analysis of Medicare reimbursement from 2007 to 2022 demonstrated that reimbursement for EGD with biopsy (Current Procedural Terminology [CPT] 43239) declined by approximately 35% after inflation adjustment.^21^ This decline has occurred despite increasing emphasis on careful mucosal inspection, systematic biopsy protocols, and longer examination times required for effective detection of gastric premalignant lesions and early cancer.

In the United States, there is currently no dedicated billing code for GC screening EGD performed in asymptomatic high-risk individuals. As a result, GC screening is typically billed under standard diagnostic EGD codes, which do not account for extended inspection time, systematic biopsy protocols, or risk stratification. The absence of a defined billing code creates a structural disincentive for endoscopists to spend the additional time required for meticulous gastric inspection, particularly in busy clinical settings.

Establishing a dedicated billing pathway for GC screening EGD, with appropriate relative value units reflecting the additional time required for systematic mucosal evaluation and targeted biopsies, may better align reimbursement with evidence-based screening practices and incentivize thorough examinations in high-risk populations. In addition, integrating GC screening EGD into existing colorectal cancer screening programs for high-risk populations may represent a potential policy approach. Bundling EGD with an already scheduled screening colonoscopy could allow GC screening to be performed with minimal additional resource utilization, resulting in only a modest incremental cost while potentially improving early detection in high-risk individuals. Given emerging cost-effectiveness data from both U.S. and European studies, such bundled screening strategies may represent a scalable approach to improving GC detection while optimizing health care resource utilization.

## HEREDITARY GC SYNDROMES AND SURVEILLANCE CHALLENGES

Another population in which the limitations of the current billing infrastructure become evident is patients with hereditary cancer syndromes. Hereditary diffuse GC, most commonly associated with germline *CDH1* mutations, carries a markedly elevated lifetime risk of GC, with estimates ranging from ∼70% to 80% in both men and women.^22,23^ Consequently, the International GC Linkage Consortium recommends prophylactic gastrectomy for individuals with pathogenic *CDH1* mutations, typically between the ages of 20 and 30 years.^22^ However, in patients who elect to defer surgery, intensive endoscopic surveillance with systematic random biopsies has been described as an acceptable alternative in the literature.^24^ In addition, endoscopic surveillance is recommended in other hereditary cancer syndromes, including Lynch syndrome and familial adenomatous polyposis, where periodic EGD may facilitate early detection of gastric neoplasia or premalignant lesions.^25,26^ These surveillance examinations often require longer procedure times and systematic biopsy protocols to ensure adequate mucosal evaluation. Despite this complexity, there are currently no dedicated CPT codes for endoscopic screening and surveillance of hereditary GCs, and these procedures are typically reimbursed under standard diagnostic EGD codes.

In conclusion, addressing GC outcomes in the United States will require more than heightened awareness or conditional guideline recommendations. It will require a reimbursement infrastructure that aligns with clinical evidence, supports risk-based screening, and recognizes the value of prevention and early detection. The development of dedicated billing pathway for GC screening and surveillance and continued advocacy through professional societies such as the American College of Gastroenterology (ACG) and the American Society of Gastrointestinal Endoscopy (ASGE) represent critical steps forward.

## DISCLOSURES

Author contributions: DS Dahiya is the article guarantor.

Financial disclosure: None to report.

## ABBREVIATIONS:

ACG: American College of Gastroenterology
AGA: American Gastroenterological Association
ASGE: American Society for Gastrointestinal Endoscopy
CPT: current procedural terminology
EBR: endoscopic biopsy rate
EGD: esophagogastroduodenoscopy
GC: gastric cancer
GI: gastrointestinal
GIM: gastric intestinal metaplasia
*H. pylori*: *Helicobacter pylori*
HDGC: hereditary diffuse gastric cancer
IGCLC: International Gastric Cancer Linkage Consortium
QALY: quality-adjusted life year
RVU: relative value unit
U.S.: United States
